# Copper: from neurotransmission to neuroproteostasis

**DOI:** 10.3389/fnagi.2014.00143

**Published:** 2014-07-03

**Authors:** Carlos M. Opazo, Mark A. Greenough, Ashley I. Bush

**Affiliations:** Oxidation Biology Laboratory, The Florey Institute of Neuroscience and Mental Health, The University of MelbourneMelbourne, VIC, Australia

**Keywords:** copper, E-ligases, neurotransmission, proteasome, synaptic activity, ubiquitination, hippocampal neurons, AMPA

## Abstract

Copper is critical for the Central Nervous System (CNS) development and function. In particular, different studies have shown the effect of copper at brain synapses, where it inhibits Long Term Potentation (LTP) and receptor pharmacology. Paradoxically, according to recent studies copper is required for a normal LTP response. Copper is released at the synaptic cleft, where it blocks glutamate receptors, which explain its blocking effects on excitatory neurotransmission. Our results indicate that copper also enhances neurotransmission through the accumulation of PSD95 protein, which increase the levels of α-amino-3-hydroxy-5-methyl-4-isoxazolepropionic acid (AMPA) receptors located at the plasma membrane of the post-synaptic density. Thus, our findings represent a novel mechanism for the action of copper, which may have implications for the neurophysiology and neuropathology of the CNS. These data indicate that synaptic configuration is sensitive to transient changes in transition metal homeostasis. Our results suggest that copper increases GluA1 subunit levels of the AMPA receptor through the anchorage of AMPA receptors to the plasma membrane as a result of PSD-95 accumulation. Here, we will review the role of copper on neurotransmission of CNS neurons. In addition, we will discuss the potential mechanisms by which copper could modulate neuronal proteostasis (“neuroproteostasis”) in the CNS with focus in the Ubiquitin Proteasome System (UPS), which is particularly relevant to neurological disorders such as Alzheimer’s disease (AD) where copper and protein dyshomeostasis may contribute to neurodegeneration. An understanding of these mechanisms may ultimately lead to the development of novel therapeutic approaches to control metal and synaptic alterations observed in AD patients.

## Introduction

Copper has a role in different pathways on the Central Nervous System (CNS; Linder and Hazegh-Azam, 1996; Gaier et al., 2013). It is essential for brain function since its deficiency lead to brain abnormalities and defects in brain development (Everson et al., 1967; Scheiber et al., 2014). This is highlighted by Menkes disease, an inherited disorder of intestinal copper absorption that has a multitude of symptoms including severe neurological degeneration and typically results in death by the age of five (Tümer and Møller, 2010). Bioavailable copper is found in the cerebrospinal fluid (~70 μM) as well as in the brain extracellular space (~1 μM) (Stuerenburg, 2000).

Copper concentration varies by brain region and becomes progressively detectable during postnatal stages (Kozma and Ferke, 1979). In rat brain, copper rapidly increases between day 5–14 postnatal (Tarohda et al., 2004) and is concentrated in the neuropil, where is mainly found on presynaptic boutons that innervate postsynaptic densities of locus ceruleus neurons (Sato et al., 1994). In effect, copper seems to be concentrated in synaptosomes and synaptic vesicles relative to magnesium, zinc and iron (Colburn and Maas, 1965). In synaptic vesicles, copper can form complexes with neurotransmitters. For example, copper can form ternary complexes with Adenosine triphosphate (ATP) and norepinephrine (Colburn and Maas, 1965). Interestingly, uptake of norepinephrine is inhibited by ethylenediamine hydrochloride, indicating that copper can participate in the uptake of neurotransmitters (Colburn and Maas, 1965). It is also known that there is a reduction in dopamine associated with dietary copper deficiency in humans (Prohaska and Bailey, 1994), highlighting its role in neurotransmitter synthesis. In addition, copper might be co-ordinating with membrane constituents of synaptic vesicles and hence may play an important role in membrane structure and function. In fact, copper can form complexes with phophatidyl-L-serine and phosphatidyl inositide, which is modulated by ATP (Maas and Colburn, 1965). These early studies supported a role for copper on neurotransmission.

## Copper and synaptic function

Koefoed-Johnsen and Ussing revealed that copper converts the frog skin membrane into a structure, which becomes selectively impermeable to chloride ions (Koefoed-Johnsen and Ussing, 1958; Palmer and Andersen, 2008), suggesting that copper could modify the permeability of plasma membrane at the presynaptic or postsynaptic levels. In agreement with a role for copper on neurotransmission, copper is released from isolated rat brain cortical synaptosomes stimulated by 50 mM KCl (Kardos et al., 1989), which was corroborated in later studies using isolated guinea-pig cerebrocortical synaptosomes (Hopt et al., 2003). Moreover, glutamate receptor activation by NMDA promotes a rapid release of copper on primary hippocampal cultures (Schlief et al., 2005).

It in this regard that it has been suggested that CNS neurons possess the machinery to uptake copper and subsequently release it at the synaptic cleft (Hartter and Barnea, 1988), where it may modulate excitatory and inhibitory neurotransmission. In agreement with this, copper blocks GABAergic and AMPAergic neurotransmission when it is applied acutely on cultured rat olfactory bulb neurons (Trombley and Shepherd, 1996). It also blocks AMPAergic neurotransmission on rat cortical neurons (Weiser and Wienrich, 1996) and GABAergic neurotransmission in acutely isolated cerebellar Purkinje cells from rat (Sharonova et al., 1998), indicating that copper modulates neurotransmission of different CNS neurons in a similar fashion. Interestingly, a recent study indicated that extra-synaptic GABA receptors are susceptible to copper modulation (McGee et al., 2013), suggesting that a spillover of copper at extrasynaptic sites, after it is released at the synaptic space, can regulate extra-synaptic receptors.

Studies performed using rat brain slices have demonstrated the acute inhibitory effect of copper on Long Term Potentation (LTP; Doreulee et al., 1997; Goldschmith et al., 2005; Leiva et al., 2009), which can be related to the effect of copper on NMDA receptor pharmacology acting as a non-competitive antagonist (Vlachová et al., 1996). Moreover, copper can inhibit LTP in the CA3 region of mouse hippocampus by a NMDA receptor-independent mechanism (Salazar-Weber and Smith, 2011). However, recent studies indicate that the role of copper on LTP regulation is more complex, because copper has shown to be required for a normal LTP response (Gaier et al., 2013, 2014a,b).

Therefore, until a few years ago, copper was considered as a negative modulator of neurotransmission. However, the effect of copper on synaptic activity has been recently evaluated in more detail (Peters et al., 2011). We have studied the synaptic activity of primary cultures of rat hippocampal neurons in the presence of copper (up to 10 μM) at different timepoints (0, 3 and 24 h). As previously described, copper blocks neurotransmission when is acutely applied to the neurons. However, after 3 h of exposure, copper promotes an increase in the AMPAergic neurotransmission, which correlates with the accumulation of PSD95 protein and with a concomitant clustering of α-amino-3-hydroxy-5-methyl-4-isoxazolepropionic acid (AMPA) receptors at the plasma membrane. Therefore, copper regulates neurotransmission by a novel biphasic mechanism, which have implications for the neurophysiology and neuropathology of the CNS. This biphasic response to copper may be not limited to hippocampal cultures and AMPAergic neurotransmission, because copper can promote a similar biphasic response on NMDA currents in cultured neonatal rat cerebellum granule cells (Marchetti et al., 2014).

Primary hippocampal neurons (10–14 DIV) treated with copper (CuCl_2_; up to 10 μM) for a short period of time (3 h) display a significant increase either in the frequency, amplitude and the time constants of synaptic events. In addition, copper increases the frequency of calcium transients, which correlated with the increase in the frequency of miniature synaptic currents, supporting the role of copper as a neurotransmission enhancer (Peters et al., 2011). Under these conditions both AMPAergic and GABAergic neurotransmission are enhanced in neurons exposed to copper. All neurotransmission parameters including amplitude, frequency and time constant of AMPA receptors were modified. However, while both the amplitude and the frequency of miniature synaptic currents were enhanced, the time constant of AMPA miniature events was decreased in copper-treated neurons (Peters et al., 2011). Interestingly, copper-treated neurons displayed changes only in the amplitude and time constant parameters of GABAergic neurotransmission. In this case, both amplitude and time constant of GABA synaptic events were increased in neurons exposed to copper. The increase in the amplitude of GABAergic currents was accompanied by an increase in GABA_A_ receptors immunostaining. Therefore, both AMPAergic and GABAergic neurotransmission contribute to the changes in total synaptic activity induced by copper.

The fact that copper-treated neurons displayed an increase in amplitude of miniature synaptic currents may be explained by an increase in the levels of receptors located post-synaptically. In this sense, both the postsynaptic clusters of GABA_A_ and AMPA receptors, located apparently at the plasma membrane, are increased after 3 h treatment with copper. GluA1 and GluA2 staining were significantly increased at MAP2-positive dendritic zones of copper-treated neurons. However, total levels of GluA1 and GluA2 subunits of the AMPA receptor did not change. Moreover, neurons exposed to copper for 3 h were more sensitive to AMPA compared to neurons incubated in basal conditions. Interestingly, the desensitization of AMPA receptors was slower in neurons exposed to copper as indicated by the values for peak/plateau of the AMPA evoked currents. In summary, neurons behave differently to copper under acute vs. prolonged incubation time, through mechanisms that may involve homeostatic or anti-homeostatic mechanisms (Carrasco et al., 2007).

**Figure 1:**
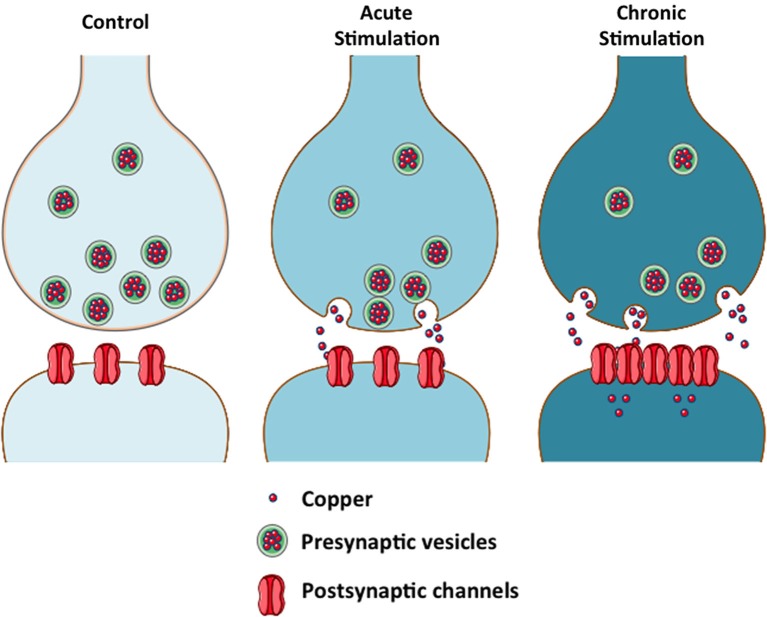
**Copper modulates neurotransmission by a biphasic mechanism**. The scheme depicts the effect of copper on neurotransmission in acute and chronic conditions. Copper acts as a channel blocker under acute conditions. Sustained release of copper from the presynaptic vesicles to the synaptic cleft will lead to an increase in intracellular copper at the postsynaptic neuron, where copper might regulate the levels of scaffolding proteins that modulate the localization of channels at the plasma membrane (Peters et al., 2011).

Thus we propose that copper enhances AMPAergic neurotransmission by promoting the clustering of AMPA receptors at the plasma membrane (See Figure 1), in a different fashion to CTR1 (copper transporter 1), the major copper uptake protein that is endocytosed and subsequently degraded in the presence of copper (Nose et al., 2010).

**Figure 2:**
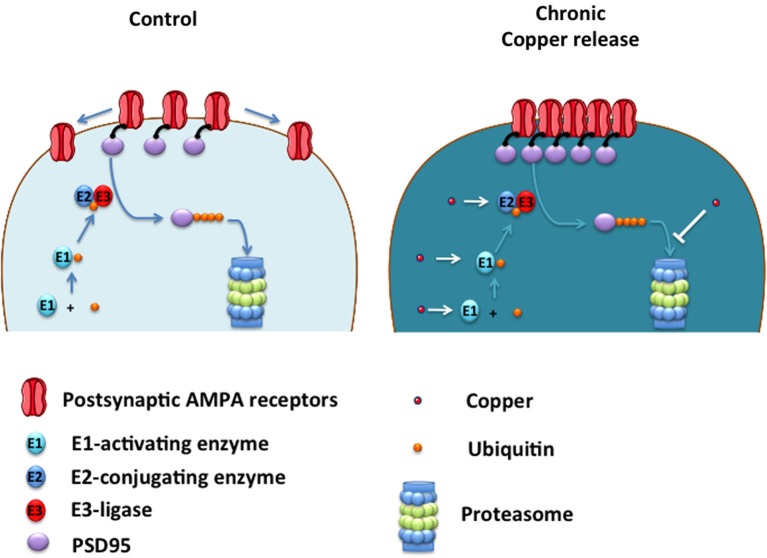
**Proposed model of how copper enhances neurotransmission by acting on UPS**. Ubiquitin (Ub) is sequentially transferred from E1-activating enzyme to E2-conjugating enzyme, and then transferred to PSD95 by the action of an E3-ligase, which lead to PSD95 degradation into the Proteasome. Under low copper levels (control), small number of AMPA receptors are located at the plasma membrane. Under chronic copper release, levels of PSD95 are increased leading to the clustering of AMPA receptors located at the plasma membrane. Copper may promote the ubiquitination of PSD95 by acting as a cofactor of the E1-E2-E3 enzymes, promoting the ubiquitination of PSD95 and a subsequent saturation of the proteasome, slowing down PSD95 degradation leading to AMPA receptor clustering at the plasma membrane. Alternatively, copper can inhibit the proteasome directly impeding PSD95 degradation and promoting the formation of AMPA clusters at the postsynaptic membrane with a concomitant enhancement of AMPAergic neurotransmission.

The clustering of AMPA receptors to the plasma membrane was accompanied by an increase in PSD95, a critical scaffolding protein for the anchoring of AMPA receptors to the cell surface (Colledge et al., 2003). Therefore, copper-treated neurons accumulate PSD95 by a mechanism that could involve a direct interaction of PSD95 with copper that increases protein stability or decreasing its degradation by the proteasome (Colledge et al., 2003; See Figure 2).

Overall, these results indicate that neurons exposed to a copper-enriched media display a more efficient neurotransmission, which correlates with changes in AMPA receptor localization/clustering and increase in the levels of PSD95. Our results indicate that copper enhances neurotransmission by changing the neuronal protein configuration and not simply due by changes in receptor pharmacology. We propose that copper might affect the neuroproteostasis of CNS neurons that lead to changes in neuronal excitability.

In support of this hypothesis, the effect of copper (3 h) on neurotransmission seemed to be unrelated to an homestotaic response resulting from the inhibition of AMPAergic neurotransmission, because after blockade of AMPA receptors for 3 h with 6-cyano-7-nitroquinoxaline-2,3-dione (CNQX), which is a specific and potent antagonist of AMPA currents, did not change any parameter of the total miniature synaptic currents, indicating that at this time frame a compensatory mechanism is not resulting for receptor blockade. Therefore, the mechanism behind the effect of copper on this neuronal network might involve intracellular changes not related to AMPA receptor blockade. Moreover, the effect of copper on neurotransmission is a transient effect because the synaptic activity returned to the control levels after 24 h of incubation, indicating a homeostatic regulation.

These studies indicate that copper might induce biphasic effects on neurotransmission, suggesting that a fine regulation of this essential metal is probably needed by neuronal cells to maintain adequate synaptic function. A failure in this copper-dependent synaptic regulation can be relevant to brain conditions where the depletion in brain copper levels are associated to a cognitive decline such as Alzheimer’s Disease (AD; Schrag et al., 2011). Therefore, further studies are required to better understand the molecular pathways that are affected by copper in living neurons. The data reviewed here indicates that copper can regulate the levels of PSD95, an intracellular scaffolding protein that modulate AMPAergic neurotransmission. Because PSD95 is degraded by the ubiquitin proteasome system (UPS; Colledge et al., 2003), copper might regulate PSD95 levels by targeting components of the UPS critical for PSD95 degradation (Figure 2).

## Copper and ubiquitin proteasome system

Ubiquitin plays a critical role in protein degradation driven by 26S Proteasome (Hershko and Ciechanover, 1998). The UPS is a major pathway by which cells remove normal proteins and abnormally folded normal or mutant, cytoplasmic and membrane proteins (Tai and Schuman, 2008). Thus, an important number of cellular processes are regulated by ubiquitin-mediated signaling events (Hicke and Dunn, 2003) and UPS dysfunction is associated with neurodegenerative disorders (Rubinsztein, 2006) that are characterized by a metal dyshomeostasis, such as AD and Parkinson’s disease (PD; Bush, 2003). The connection with ubiquitin and the proteasome is physiologically hierarchical and can be biochemically dissected in two main components (Ciehanover et al., 1978). Proteins are first ubiquitinated (Hershko et al., 1983) and then recognized by the 26S Proteasome for degradation (Deveraux et al., 1994). The key enzymes that regulate protein ubiquitination are E-activating, E-conjugating and E-ligases (Ciechanover et al., 1982; Hershko et al., 1983). Protein ubiquitination begins with the fast formation of a thiol-ester linkage between the C-terminus of ubiquitin and the active site cysteine of the ubiquitin-activating enzyme (E1) (Hershko et al., 1981; Pierce et al., 2009). This initial step requires ATP and ionic cofactors, including Mg^2+^ and an unknown metal ion (Ciechanover et al., 1982). The absence of these ionic factors stop ubiquitination. Thus, copper could act as “the unknown metal ion” in this enzymatic reaction. Further studies are needed to validate this possibility. Ubiquitin is then transferred to an ubiquitin conjugating enzyme (E2) (Hershko et al., 1983) to form a catalytically activated intermediate such as the UbcH5b Ub (Sakata et al., 2010). UbcH5b is one of the E2 enzymes that has been demonstrated to form polyubiquitin chains in cooperation with several E3 enzymes (Wu et al., 2003; Brzovic et al., 2006; Sakata et al., 2007; Windheim et al., 2008). These E3 ligases interact with UbcH5b-Ub intermediate, catalyzing the formation of an isopeptide bond between the C-terminal residue of ubiquitin (G76) and a lysine located either on a target protein or on the lysine (usually K48 for degradation) of the most peripheral ubiquitin tagged to a protein (Sakata et al., 2010), which then directs it to the 26S proteasome for degradation (Pickart, 2000).

There are several studies that connect the UPS to transition metals. For example, Kojima’s group characterized the* in vitro* interaction between ubiquitin and copper by using electron paramagnetic resonance (EPR) approximation (Nomura et al., 2004). This study strongly suggested that Cu^2+^, as a part of one metal complex, is coordinated by ubiquitin with the participation of a histidine residue (his-68). Other paramagnetic metals, such as Mn^2+^ and Gd^3+^, did not coordinate specifically to his-68 present in ubiquitin sequence (Nomura et al., 2004). In fact, ubiquitin is retained to immobilized metal ion affinity chromatography (IMAC) resins complexed to Cu^2+^ (Hemdan et al., 1989), where his-68 is critical for this binding. Interestingly, when his-68, located at the surface of ubiquitin (Sloper-Mould et al., 2001) is replaced with another residue, the ubiquitination process (Ecker et al., 1987) or cell growth is altered (Sloper-Mould et al., 2001). All this data suggests that copper might participate upstream in the regulation of UPS. However, copper complexed to some chelators inhibit the proteasome for unknown mechanism (Ding and Lind, 2009), suggesting the possibility that copper can regulate the UPS at different levels (Figure 2).

Metalloproteins are part of the UPS, acting as E3-Ring ligases or deubiquitinases (Joazeiro and Weissman, 2000; Yao and Cohen, 2002), but it is unclear if transition metals can participate upstream in the regulation of the first steps of ubiquitination. Downstream of the UPS, proteasome activity is inhibited by copper at milimolar concentration (Amici et al., 2002) and some copper-chelator complexes can also inhibit proteasome activity (Ding and Lind, 2009). Zinc is critical for the activity of E3-Ring ligases and RPN11 deubiquitinase (Joazeiro and Weissman, 2000; Yao and Cohen, 2002). Moreover, metal response to cadmium toxicity in yeast involves the inactivation of Skp1-Cullin1-F-box (SCF) ligases complexes (Yen et al., 2005) and also the activation of UPS (Jungmann et al., 1993). However, cadmium can also induce the accumulation of ubiquitinated proteins by an oxidative mechanism that leads to neurotoxicity (Figueiredo-Pereira et al., 1998). Interestingly, iron can accelerate the degradation of proteins into the proteasome by inducing oxidative modifications in the targeted protein (Iwai et al., 1998). Moreover, it can regulate the ligase activity of SKP1-CUL1-FBXL5 protein complex (Salahudeen et al., 2009; Vashisht et al., 2009). Therefore, metals such as copper may activate or inactivate early steps of ubiquitination. Interestingly, copper is specifically coordinated by ubiquitin (Hemdan et al., 1989; Nomura et al., 2004), indicating that this metal can act at early stages of ubiquitination. In fact, copper is required for Ctr1 poly-ubiquitination and subsequent degradation by a mechanism that requires the presence of the copper chaperone Atox1 (Safaei et al., 2009). Moreover, Clioquinol, a copper chelator with moderate affinity, can inhibit *in vitro* ubiquitination of Hypoxia-inducible Factor-1α (Choi et al., 2006), indicating that copper may participate as a cofactor in ubiquitination.

## Conclusion

Inherited disorders of Cu metabolism, such as Menkes and Wilson’s disease display complex neurodegenerative features, which highlight the importance of copper homeostasis (Tümer and Møller, 2010). Moreover, micromolar concentrations of copper (up to 400 μM) are present in senile plaques in AD brains (Lovell et al., 1998), which could be a source of copper for the neurons surrounding these pathological structures. Brain copper deficiency is a characteristic feature of Menkes disease, which affects brain physiology, since patients display gray matter degeneration, hippocampal neuronal loss and Purkinje cell abnormalities (Okeda et al., 1991). AD is another brain pathology characterized by neurodegeneration that produces a broad spectrum of symptoms that have been linked to copper brain depletion since cupro-proproteins such as ceruloplasmin are decreased (Connor et al., 1993; Bush, 2003) or less active as observed for superoxide dismutase 1 (Omar et al., 1999; Maynard et al., 2005). Currently, the relationship between copper and neuroproteostasis within the CNS in health and pathological conditions is poorly understood. Hence, further studies are required to determine how neuronal excitability is linked to changes in synaptic proteins promoted by copper (Gaier et al., 2013). The studies described here provide a new perspective on how copper can regulate the communication between neurons by modifying the protein configuration and strength of neurotransmission within the CNS.

## Conflict of interest statement

The authors declare that the research was conducted in the absence of any commercial or financial relationships that could be construed as a potential conflict of interest.

## Abbreviations

AD: Alzheimer’s disease
AMPA: α-amino-3-hydroxy-5-methyl-4-isoxazolepropionic acid
ATP: Adenosine triphosphate
CNQX: 6-cyano-7-nitroquinoxaline-2,3-dione
CTR1: copper transporter 1
GABA: γ-aminobutyric acid
LTP: long term potentiation
UPS: Ubiquitin Proteasome System.
